# Effects of Electroacupuncture on the Correlation between Serum and Central Immunity in AD Model Animals

**DOI:** 10.1155/2022/3478847

**Published:** 2022-09-13

**Authors:** Jing Jiang, Zidong Wang, Ruxia Yu, Jiayi Yang, Huiling Tian, Hao Liu, Shun Wang, Zhigang Li, Xiaoshu Zhu

**Affiliations:** ^1^Beijing University of Chinese Medicine, School of Nursing, Beijing, China; ^2^Western Sydney University, School of Health Sciences, Sydney, Australia

## Abstract

**Objective:**

The goal was to investigate the connection between neuroinflammation in the brain and serum inflammatory markers as Alzheimer's disease progressed. We also sought to determine whether electroacupuncture had an effect on inflammatory markers found in blood and other brain regions.

**Methods:**

As an animal model for AD, we used senescence-accelerated mouse prone 8 (SAMP8) mice. To examine the effects and probable mechanism of electroacupuncture, we used HE staining, immunofluorescence staining, western blotting, and enzyme-linked immunosorbent assay.

**Results:**

Electroacupuncture therapy protected neurons, significantly downregulated the Iba-1 level in the hippocampus (*p* value was 0.003), frontal lobe cortex (*p* value was 0.042), and temporal lobe cortex (*p* value was 0.013) of the AD animal model, all of which had significantly lower levels of IL-6 (*p* value was 0.001), IL-1*β* (*p* value was 0.001), and TNF-*α* (*p* value was 0.001) in their serum.

**Conclusion:**

The amounts of IL-6, IL-1*β*, and TNF-*α* detected in the serum were strongly linked to the levels discovered in the hippocampus and the frontal lobes of the brain, respectively. A better understanding of the electroacupuncture process as well as the course of Alzheimer's disease and the therapeutic benefits of electroacupuncture may be gained by using biomarkers such as serum inflammatory marker biomarkers.

## 1. Introduction

One of the progressive neurodegenerative diseases afflicting individuals all over the world is Alzheimer's disease (AD) [1, 2]. Many kinds of therapeutics have been investigated for AD patients, but there is still no efficacy treatment to stop and reverse the progress of this disease [3]. Even though intracellular hyperphosphorylated tau protein tangles and extracellular senile plaques with aggregated amyloid beta (A*β*) in the cerebrum are pathogenic features of Alzheimer's disease [4, 5], the neuroinflammatory response associated with neuronal dysfunction in the progression of AD is gaining increasing attention [6–8]. It was revealed that prolonged activation of microglia cells and the resulting overproduction of inflammatory factors, including IL-6, IL-1*β*, and TNF-*α*, were significant contributions to the development of Alzheimer's disease. As a consequence, microglia activation and accumulation of inflammatory markers in the different regions of brain were identified as the third pathogenic characteristic of the AD process. Certain studies have shown that acupuncture treatment reduces inflammation and protects neurons in the brain [9]. Acupuncture therapy has also been shown to reduce inflammation and protect neurons in the brain by certain researchers [10–12].

Additionally, we found that electroacupuncture might enhance SAMP8 mice's spatial learning and memory capacities [13, 14], increase the glucose metabolism level of neurons in the different brain regions [15, 16], and decrease the neuroinflammatory response by upregulating the triggering receptor expressed on myeloid cells 2 (TREM2) in hippocampus [17–19] based on previous research by our research team. Using these data, we were able to validate the effects of electroacupuncture intervention of Alzheimer's disease as well as to investigate its possible mechanism in terms of the central nervous system's inflammatory response. Slow and often accompanied by peripheral and central symptoms are common features of Alzheimer's disease development. As a means to better understand Alzheimer's and achieve early diagnosis and treatment, scientists focused on discovering biomarkers for the illness in cerebrospinal fluid [20, 21], serum [22–24], body fluid [25, 26], feces, and urine [27]. In different clinical research, some proinflammatory factors (such as IL-6, IL-1*β*, and TNF-*α*) were reported to be overexpressed in patients with Alzheimer's disease and elderly people with mild cognitive impairment [28–31]. A rising serum IL-1*β* level was found to be a stage marker of continuous brain impairment throughout the continuum between healthy aging and AD patients in a thorough investigation and meta-analysis [32]. These findings provide evidence of a connection between neuroinflammation in the central nervous system disease (such as AD) and inflammatory reaction in the body.

We wanted to see if there was a connection between the inflammatory markers in the serum and the neuroinflammation that was occurring in the various regions of brain as Alzheimer's disease processed. The influence of electroacupuncture on inflammatory markers both in the blood and in a variety of regions of the brain was another objective of our research.

## 2. Methods

### 2.1. Animals and Ethics Statement

The SPF (Beijing) Biotechnology Co., Ltd. furnished SAMP8 mice and mice having the same genetic background and antiaging properties (senescence-accelerated mouse resistant 1, SAMR1). They weighed 28.0 ± 2.0 g and were 7 months old. Mice were housed at the Animal Experimentation Center with SPF-level barrier, where they were provided with pellet food and kept at a temperature of 22°C at all times, with a consistent cycle of 12 hours of darkness and light. Before the experiment, all mice spent five days acclimating to their habitat.

The experimental approach that was used in this research was given approval by the ethical committee for animal experimentation. All operations were carried out in accordance with the Animal Research: Reporting In Vivo Experiments (ARRIVE) standards. Each researcher in this article has obtained accreditation from the animal experimental center.

### 2.2. Grouping and Treatment

A random selection was used to assign ten SAMP8 male mice to each of the following three groups (*n* = 10 each): the electroacupuncture group (also known as the EA group), donepezil group, and Alzheimer's disease model group (AD group). A normal control group consisting of ten male SAMR1 mice was used in the study. Mice were given mice hydrochloride tablets of donepezil (Eisai China Inc., H20050978) at a dosage of 0.65 *μ*g/g through oral gavage in the donepezil group. Two acupuncture needles (0.25 mm*∗*13 mm; Beijing Huatuo Medical Instrument Co., Ltd) were transverse incisions at EX-HN3 (Yintang) and GV20 (Baihui) used in the EA group. A piece of tape was used to secure the needles to the respective acupoints. The HANS-LH200/100B EA device (Beijing Xingyu Hongye Trading Co., Ltd.) was used to stimulate the needles with sparse waves at 2 Hz, 2 V, and 0.1 mA intensity. The control, donepezil, and AD groups mice were all immobile for a total of fifteen minutes throughout the experiment (without electroacupuncture treatment).

### 2.3. HE and Immunofluorescence Staining

From each group, three mice were randomly selected, and their brains were taken out and stained with hematoxylin and eosin and immunofluorescence to observe the situation of neurons and the level of microglia activation in the frontal, temporal, and hippocampus.

These tissues were dewaxed three times in xylene for five minutes each, then for five minutes each in anhydrous ethanol, 90 percent ethanol, 70 percent ethanol, and distilled water. The tissues were then dried, paraffin-embedded, and cut into slices (10 mm thick). Sections were dyed with ethanol and xylene at escalating concentrations before being dried. Each specimen's frontal lobe cortex, temporal lobe cortex, and the hippocampus (dentate gyrus) were examined using a 40x Olympus light microscope.

In different regions of the brain, immunofluorescence labeling was employed to detect indications of microglia activation. Following dewaxing and hydration, sections were treated in Triton X-100 (mass fraction 0.5 percent) for 10 minutes, blocked with bovine serum albumin BAS (mass fraction 2 percent), and finally allowed to rest at normal room temperature for one hour. Then, the sections were treated with the antibody Iba-1 (USA, Protein tech, 1 : 150) for a whole night at a temperature of 4°C, washed by PBS three times, and then combined with the FITC fluorescently tagged secondary antibody. After being dried, the slices were evaluated after being scanned with a digital pathological slice scanner (NDP), which also logged the data.

### 2.4. Western Blot Analysis

Brain and serum samples were taken from other mice in each group while they were anesthetized. An electrophoresis in which 10% of the gel was used for separation and 5% for stacking was carried out after protein extraction (SDS-PAGE). On PVDF membranes with a 0.45 *μ*m thickness, proteins were subsequently deposited. The combination of 0.1 percent Tween 20 and 5% nonfat milk in tris-buffered saline was used to block membranes (TBST). Overnight at 4°C, membranes were incubated with rabbit polyclonal Iba1 primary antibodies (1 : 1000, Proteintech). After mixing the sample and letting it sit for an hour at room temperature, rabbit anti-mouse H&L IgG secondary antibodies diluted at 1 : 2000 ratio were added. Following the developing and fixing steps, an Immobilon western chemiluminescent horseradish peroxidase (HRP) substrate was used to expose the X-ray film in the dark. Quantity One was responsible for the scanning and analysis, and the results included a comparison of the levels of the Iba1 expression between the groups after calibration to the expression of GAPDH.

### 2.5. ELISA Analysis

Proinflammatory IL-6 (RayBiotech, KE10007), IL-1 (RayBiotech, KE10003), and TNF (Raybiotech, KE10002) levels in serum, frontal lobe, and hippocampus were measured using the standard ELISA technique (dilution concentration 1 : 5). The particular stages were carried out precisely as specified in the kit's instructions.

### 2.6. Statistical Examination

For statistical analysis, all data were examined by Statistical Package for the Social Sciences (SPSS) version 22.0 (SPSS Inc.). A value of 0.05 was chosen to serve as the significance criterion for each comparison. The statistical significance of intergroup differences was determined using the *t*-test for independent samples for continuous variables and Pearson's chi-squared test for categorical variables. The expression levels of IL-6, IL-1*β*, TNF-*α*, and Iba-1 were compared using one-way analysis of variance (ANOVA) and Games–Howell post hoc analysis after Levene's test revealed that the variances were not equal. Each variable's Spearman correlation coefficient was calculated. In a multivariate regression analysis, Iba-1 levels in different brain regions served as the dependent variable, while other inflammatory factors served as the explanatory variables.

## 3. Results

### 3.1. In Several Parts of the Brain, EA Defends Neurons and Prevents Microglia from Becoming Active

**Figure 1:**
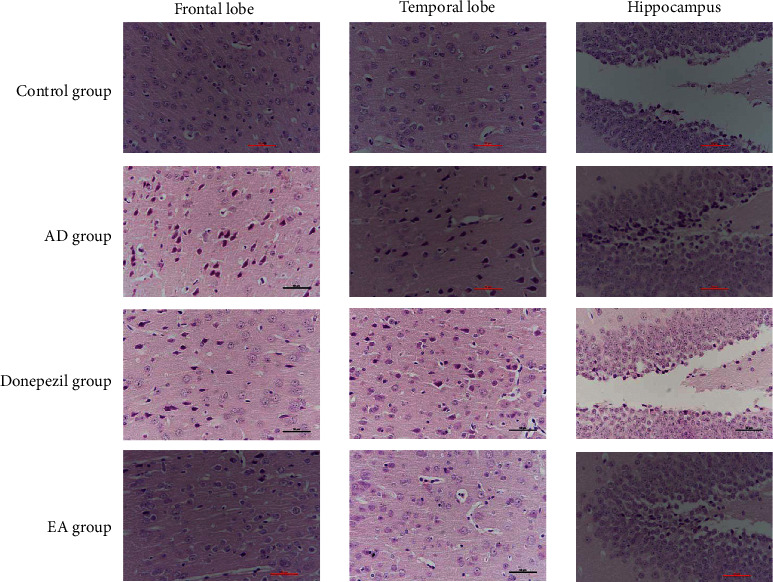
Results of HE staining. (HE staining of each group in the frontal lobe cortex, temporal lobe cortex, and dentate gyrus of hippocampus, ^*∗*^400).

The HE staining of four different groups is shown in Figure 1. These groups were located in the frontal lobe cortex and temporal lobe cortex of the hippocampus. In these three regions of the brain, the neurons that were clear-dyed and had kernels that could be identified were aligned in orderly rows in the mice that were in the normal control group. The neurons belonging to the AD group, on the other hand, revealed irregular and dispersed morphology as well as nuclear pyknosis. In comparison with the neurons in the AD group, those in EA and donepezil groups had much less nuclear condensation and seemed to be neatly aligned in rows. Aside from that, tissue samples collected from the EA group matched the control group's specimens the most closely. It has been shown that electroacupuncture may partly maintain neurons in hippocampus as well as in the frontal and temporal lobe cortex.

**Figure 2:**
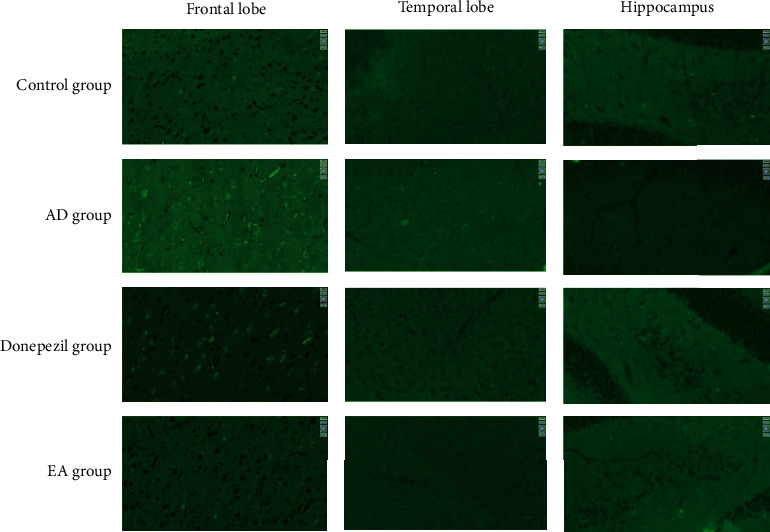
Results of immunofluorescence staining (immunofluorescence staining of each group in the frontal lobe cortex, temporal lobe cortex, and dentate gyrus of hippocampus, ^*∗*^400. Green light was labeled the expression of Iba-1).

**Figure 3:**
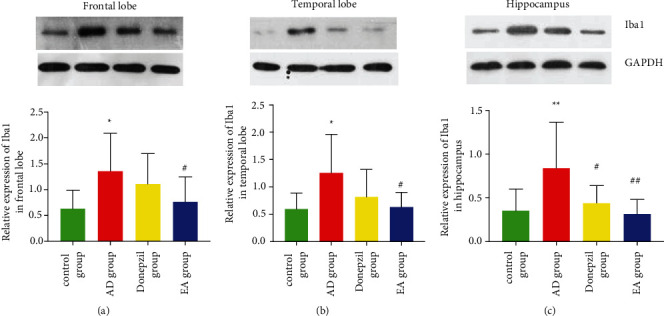
Results of western blotting test (Iba-1 was expressed differently by each group of microglia in the frontal, temporal, and hippocampal lobes). (a) Frontal lobe. (b) Temporal lobe. (c) Hippocampus. ^*∗*^Compared with the control group, *p* < 0.05; ^*∗∗*^compared with the control group *p* < 0.01; ^#^compared with the AD group, *p* < 0.05; ^##^compared with the AD group, *p* < 0.01.

As shown in Figure 2, the frontal lobe, temporal lobe, and hippocampus exhibited significant amounts of the Iba-1 protein expression on the membrane of microglia.  Figure 3 depicts the level of Iba-1 in each group's three brain regions. In each region of the brain, the Iba-1 protein expression in AD group was considerably higher than normal control group, as indicated in the figure (*p* value in the frontal lobe cortex is 014, in the temporal lobe cortex is 0.01, and in the hippocampus is 0.005), suggesting that microglia activation was more serious in SAMP8 mice than in SAMR1 mice. The expression of Iba-1 was significantly lower in the EA group compared to the AD group in each brain region (*p* value in frontal lobe cortex is 0.042, in temporal lobe cortex is 0.013, in hippocampus is 0.003), indicating that EA treatment may inhibit microglia activation in the SAMP8 mice.  Figure 3(c) shows that donepezil could lower the expression of Iba-1 solely in the hippocampus (*p*=0.018 < 0.05).

**Figure 4:**
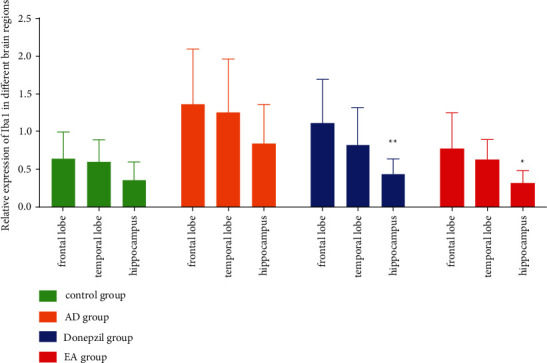
Relative expression of Iba-1 in different brain regions. ^*∗*^Compared with the frontal lobe, *p* < 0.05; ^*∗∗*^compared with the frontal lobe, *p* < 0.01.

The expression of the Iba-1 is examined in Figure 4 across different brain regions for each of the groups. The AD and control groups showed no differences in any of the areas of the brain examined. We discovered that there were significantly differences between frontal lobe and hippocampus both in EA and donepezil groups (*p* value in the donepezil group is 0.008 and in the EA group is 0.01). As a consequence of this, while investigating the relationship between serum and central immunity, we concentrated our attention on the frontal lobe and the hippocampus.

### 3.2. EA Downregulates Inflammation Factors in the Serum and Brain

By looking at inflammatory indicators, we sought to establish if there was a relationship between serum and central immunity. Consequently, we employed an ELISA to evaluate the relative proteins in serum, the frontal lobe, and the hippocampus, concentrating on the levels of IL-6, IL-1*β*, and TNF-*α*.

**Figure 5:**
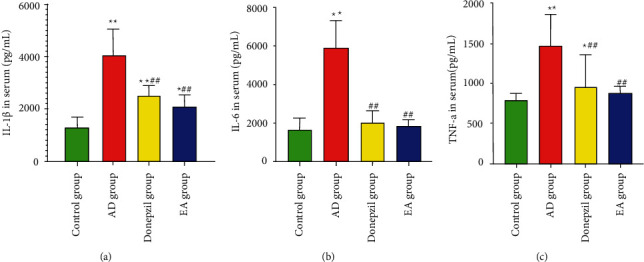
Inflammation factors in the serum of each group. (a) Comparison of IL-1*β* content in each group's serum; (b) comparison of IL-6 content in each group's serum; and (c) comparison of TNF-*α* content in each group's serum. ^*∗*^Compared with the control group, *p* < 0.05; ^*∗∗*^compared with the control group *p* < 0.01; ^#^compared with the AD group, *p* < 0.05; ^##^compared with the AD group, *p* < 0.01.

From Figure 5, the AD group exhibited considerably higher levels of IL-6, IL-1*β*, and TNF-*α* than the control group (*p* values for IL-6, IL-1*β*, and TNF-*α* were 0.001). These values were significantly reduced in the donepezil and EA groups in comparison with those in the AD group, particularly in the EA group (*p* value in the donepezil group was 0.001 for IL-1*β*, 0.001 for IL-6, and 0.001 for TNF-*α*; *p* value in the EA group was 0.001 for IL-1*β*, IL-6, and TNF-*α*). Based on these data, it seemed as if EA treatment had the potential to lower the levels of proinflammatory markers found in the serum of SAMP8 mice.

**Figure 6:**
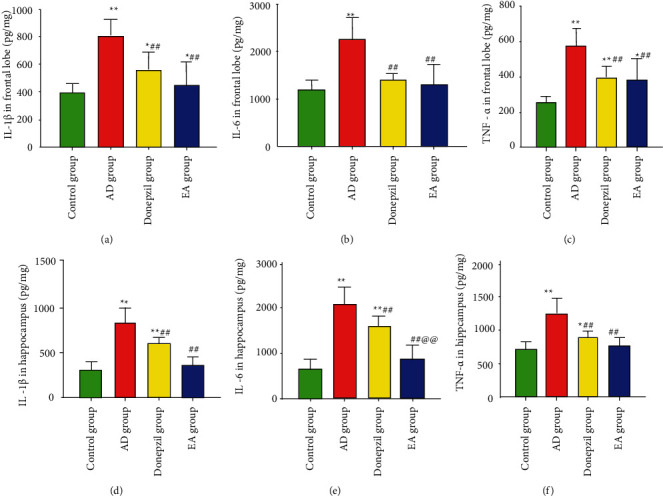
Inflammation factors in the frontal lobe cortex and hippocampus. ^*∗*^Compared with the control group, *p* < 0.05; ^*∗∗*^compared with the control group *p* < 0.01; ^#^compared with the AD group, *p* < 0.05; ^##^compared with the AD group, *p* < 0.01; ^@@^compared with the donepezil group, *p* < 0.01.

Figure 6 shows that IL-6, IL-1*β*, and TNF-*α* was considerably greater in the AD group than the control group (in the frontal lobe and hippocampus: *p* values for IL-6, IL-1*β*, and TNF-*α* were 0.001, 0.001, and 0.001, respectively). Expression of these proinflammatory markers was significantly reduced as compared to the AD group in donepezil and EA groups (*p* values were 0.003 and 0.001 for IL-1*β* at frontal lobe; *p* values were 0.002 and 0.001 for IL-1*β* at hippocampus; *p* values were both 0.001 at the frontal lobe; *p* values were 0.008 and 0.001 for IL-6 at the hippocampus; *p* values were 0.001 and 0.001 for TNF-*α* at the frontal lobe; *p* values were both 0.001 for TNF-*α* at the hippocampus). EA exhibited lower amounts of IL-6 protein in the hippocampus than the donepezil group, as seen in Figure 6(e) (*p*=0.001 < 0.01).

### 3.3. Correlation Analysis of Serum Inflammatory Factors and Inflammatory Factors and Iba-1 in Different Brain Regions

**Figure 7:**
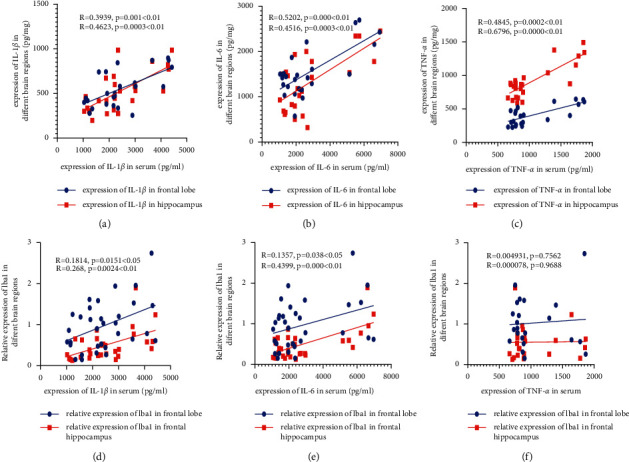
Correlation investigation of serum inflammatory markers and Iba-1 levels in various brain areas. Correlation of serum IL-1*β* level with IL-1*β* between the frontal lobe and hippocampus (a) correlation of serum IL-6 level with IL-6 between the frontal lobe and hippocampus (b) correlation of serum TNF-*α* level with TNF-*α* between the frontal lobe and hippocampus (c) correlation of serum IL-1*β* level with Iba-1 between the frontal lobe and hippocampus (d) correlation of serum IL-6 level with Iba-1 between the frontal lobe and hippocampus (e) correlation of serum TNF-*α* level with Iba-1 between the frontal lobe and hippocampus (f) Correlation analysis was evaluated using the Person correlation method (*R* value and *p* value were given).

This proinflammatory marker Iba-1 was shown to be associated with increases in serum levels of interleukin-6, interleukin-1 beta, and tumor necrosis factor-alpha. As indicated in Figure 7, there was a favorable association between the serum levels of IL-6, IL-1*β*, and TNF-*α* and the frontal lobe and hippocampal concentrations of these cytokines. According to these results, a rise in the levels of IL-6, IL-1*β*, and TNF-*α* found in the serum was associated with modifications in these proinflammatory markers found in the frontal lobe and the hippocampus.

According to these results, a rise in blood levels of IL-6, IL-1*β*, and TNF-*α* was associated with changes in these proinflammatory markers in the frontal lobe and the hippocampus. There was no significant link between the blood TNF-*α* level and Iba-1 in either frontal lobe or hippocampus; however, there was a positive connection between serum IL-1*β* and IL-6 levels and Iba-1 levels in the frontal lobe and the hippocampus (Figures 6(d)–6(f)). Increased IL-1*β* and IL-6 serum levels were shown to be associated with activation of microglia in the frontal lobe and hippocampus through raised Iba-1 levels, according to these findings.

## 4. Discussion

Given its efficacy and absence of side effects, electroacupuncture (or acupuncture), a nonpharmacologic therapeutic procedure that stimulates acupoints, has been thoroughly researched and used to treat Alzheimer's disease [33]. Acupuncture therapy improved cognitive function in patients with Alzheimer's disease and elderly people with mild cognitive impairment, according to clinical studies and systematic reviews [34–38]. Animal studies have contributed to the discovery of the mechanism by which electroacupuncture protects neurons in Alzheimer's disease. Recent investigations have demonstrated that electroacupuncture can control the microglia activation [39] and reduce the production of neuroinflammatory markers of brain [40, 41]. However, the research above mostly focused on the hippocampus, with no evidence of electroacupuncture intervention on neuroinflammatory factors and microglia cell activation in other brain areas.

A recent comprehensive investigation found that having the apolipoprotein E (APOE) 4 allele increased the likelihood of tau protein buildup in the frontal and temporal lobes, which worsened memory loss and affected executive function, visuospatial abilities, and language [42]. We looked at the hippocampus, as well as the frontal and temporal lobes, in our study. The status of neuronal apoptosis was obvious in the three regions of AD animal models' brain, as shown in Figure 1. Furthermore, as seen in Figures 2 and 3, microglia activation was observed in the abovementioned locations. Additionally, we examined the relative expression of Iba-1, a hallmark of microglia activation, in the three sites. Surprisingly, we discovered that the Iba-1 expression was much higher in the frontal lobe than in the hippocampus, particularly in the donepezil and EA groups (Figure 4). As a result, we conclude that electroacupuncture could protect neurons by inhibiting microglia activation in the frontal lobe cortex, temporal lobe cortex, and hippocampus, and that these effects of electroacupuncture are more sensitive to the hippocampus than that of the frontal lobe.

We discovered two intriguing findings in this study's correlation analysis: (1) serum inflammatory factors and brain inflammatory factors have a positive relationship; (2) serum inflammatory factors have a positive relationship with the degree of activation of microglia in the brain.

Not only AD, many central nervous system diseases have changes in peripheral serum inflammatory factors, such as depression [43, 44], Parkinson disease [45, 46], and other kinds of dementia [47, 48]. In a recent clinical investigation concerning the content of inflammatory factors in AD patients' blood, researchers revealed that not only levels of IL-6 and TNF-*α* but also IL-4, IL-10, and CCL-2 in the serum were dramatically raised and surprisingly linked with the cognitive performance of AD patients [49]. The results of our investigation are consistent with those of the aforementioned clinical trials. Electroacupuncture has been demonstrated to improve cognitive performance in SAMP8 mice and protect neuron function in our previous studies [13–16]. Electroacupuncture's mechanism for suppressing neuroinflammatory responses and controlling microglia activation in the hippocampus was the focus of these investigations [18, 19, 50]. In this study, we revealed that acupuncture (or electroacupuncture) might affect the neuroinflammation markers, such as IL-6, IL-1*β*, and TNF-*α*, both in the hippocampus and frontal lobe. According to the findings, IL-6, IL-1*β*, and TNF-*α* were all raised both in the frontal lobe and hippocampus, and electroacupuncture was able to protect the neurons by reducing these inflammatory markers.

Iba-1, a marker of activation microglia, was widely used to identify immunopositivity microglia in a number of central nervous system disorders, such as Alzheimer's disease [51], schizophrenia and bipolar disorder [52, 53], and spinal cord injury [54, 55]. Not only did we confirm that Iba-1 was significantly expressed in SAMP8 mice but we also discovered that the expression of Iba-1 in the hippocampus and frontal lobe was proven to be positively connected to the blood levels of IL-6 and IL-1*β*. What we found in this study offer a robust evidentiary base for the relationship between the immune system in serum and the central nervous system, which could be utilized to evaluate the neuroinflammation in the brain as AD progresses by examining serum inflammatory markers. These might be applied in clinics for both diagnosis and therapy.

## 5. Conclusion

In conclusion, our current investigation shown that electroacupuncture may reduce the levels of proinflammation components in serum and various brain areas while also inhibiting microglia activation in various brain regions. It was discovered that there is a significant connection between the activation of microglia in the frontal and hippocampus regions of the brain and the blood levels of proinflammatory markers, such as IL-1*β* and IL-6. These discoveries improved our understanding of the electroacupuncture procedure and helped us use simpler-to-measure serum inflammatory markers to monitor the progress of Alzheimer's disease and indicate the effects of electroacupuncture. This study had a few limitations, and there was certainly need for more investigation. The frontal lobe and hippocampus were the only anatomical sites where we found three inflammatory markers in the serum throughout this investigation. More study and data to support these are required in order to show the connection between serum and the brain and to examine the additional electroacupuncture mechanisms.

## Data Availability

All data are included in the manuscript.
